# The effects of exercise training on insulin resistance in children and adolescents with overweight or obesity: a systematic review and meta-analysis

**DOI:** 10.3389/fendo.2023.1178376

**Published:** 2023-08-10

**Authors:** Fatemeh Kazeminasab, Fatemeh Sharafifard, Maryam Miraghajani, Nasim Behzadnejad, Sara K. Rosenkranz

**Affiliations:** ^1^ Department of Physical Education and Sport Sciences, Faculty of Humanities, University of Kashan, Kashan, Iran; ^2^ Department of Cancer Research Center, Shahid Beheshti of Medical Sciences, Tehran, Iran; ^3^ Department of Exercise Physiology, Faculty of Physical Education and Sport Sciences, University of Isfahan, Isfahan, Iran; ^4^ Department of Kinesiology and Nutrition Sciences, University of Nevada Las Vegas, Las Vegas, NV, United States

**Keywords:** exercise training, insulin resistance, children, adolescents, obesity

## Abstract

**Aim:**

The aim of present meta-analysis was to determine the effects of exercise training (Exe) on insulin resistance (IR) and body weight in children and adolescents with overweight or obesity.

**Methods:**

PubMed, Web of Science, and Scopus were searched for original articles, published through October 2022 that included exercise versus control interventions on fasting glucose, insulin, HOMA-IR, and body weight outcomes in children and adolescents with overweight or obesity. Standardized mean differences (SMD) for fasting insulin, and weighted mean differences (WMD) for fasting glucose, HOMA-IR, body weight (BW), and 95% confidence intervals were determined using random effects models.

**Results:**

Thirty-five studies comprising 1,550 children and adolescents with overweight and obesity were included in the present meta-analysis. Exercise training reduced fasting glucose (WMD=-2.52 mg/dL, p=0.001), fasting insulin (SMD=-0.77, p=0.001), HOMA-IR (WMD=-0.82, p=0.001), and BW (WMD=-1.51 kg, p=0.001), as compared to a control. Subgroup analyses showed that biological sex, intervention duration, type of exercise training, BMI percentile, and health status (with or without diagnosed condition), were sources of heterogeneity.

**Conclusion:**

Exercise training is effective for lowering fasting glucose, fasting insulin, HOMA-IR, and BW in children and adolescents with overweight or obesity and could provide an important strategy for controlling IR and related factors. With clear evidence for the effectiveness of exercise interventions in this vulnerable population, it is important to determine effective approaches for increasing exercise training in children and adolescents with overweight or obesity.

## 1 Introduction

Insulin resistance (IR) is a pathological condition underpinned by reduced glucose uptake in response to physiological insulin levels and tissue responses to insulin-mediated cellular actions (1). IR is considered to be the important pathophysiologic link between obesity/overweight and many metabolic disorders, due to the disruption of various molecular pathways (2). Although the mechanisms through which excess adipose tissue causes IR are complex and not completely understood, several major mechanisms, including increases in circulating free fatty acids (FFAs), inflammation, oxidative stress, insulin receptor mutations, endoplasmic reticulum stress, and mitochondrial dysfunction, can result in insulin-signaling suppression (3, 4). Impairment in insulin signaling underpins the future development of type 2 diabetes mellitus, hypertension, hyperuricemia, and metabolic syndrome, often placing a heavy burden on patients, families, and health care systems (2).

Obesity-induced IR in children and adolescents is of the utmost importance. These periods can be considered as critical periods of development that are associated with stable lifelong trajectories of obesity and metabolic derangement, which can impair health status into adulthood (5). Therefore, emphasis has been placed on preventative (primary and secondary) strategies, such as identifying and modifying risk factors for IR among high-risk children and adolescents, focusing on early intervention (6). Previously published intervention studies have suggested that an intensive lifestyle modification program including improved dietary intake and promoting a less sedentary lifestyle, could increase weight loss, improve insulin sensitivity, and reduce the risk of developing chronic diseases (7). However, fears related to the potential for disordered eating in this vulnerable population, have led to a larger emphasis on exercise training strategies, which have become a cornerstone of obesity-induced IR treatment, and may have benefits beyond those that can be obtained by solely focusing on dietary intake for metabolic health outcomes (7). This is particularly true when considering the trajectory of metabolic health, since in children, healthy physical activity habits help establish patterns that continue into adulthood (7).It is thus important to evaluate the role of exercise training for obesity-induced IR in children and adolescents with overweight and obesity. In this field, not all studies have reported consistent results.

A recent meta-analysis published in 2021 (8), which assessed the effects of exercise interventions in children and adolescents with overweight and obesity on the risk factors of metabolic disorders, emphasized the importance of aerobic exercise to reduce fasting insulin, body mass index, and % body fat. However, insulin resistance did not change significantly. Another meta-analysis showed no significant differences in HOMA-IR, blood glucose, nor body weight following a high-intensity interval training intervention compared with moderate intensity training in children and adolescents with obesity (9). Data from another meta-analysis published in 2016 (10) which evaluated the effects of exercise training on insulin resistance markers in children and adolescents with overweight or obesity supported the importance of exercise, especially aerobic training, that was associated with the reduction of fasting insulin levels and HOMA-IR. This previous meta-analysis included only 17 studies.

Thus, given the prevalence of overweight and obesity in children and adolescents, the negative consequences associated with such, the inconsistent findings of the previous reviews in this field, the current systematic review and meta-analysis was conducted to investigate the effects of exercise training when compared to a control group, on IR indices and body weight in children and adolescents with overweight or obesity. Additionally, we have included the 35 highest quality studies available, to update the current evidence and to inform future research and best practices for children and adolescents who are at risk for developing IR. This systematic review and meta-analysis sought to elucidate the potential of exercise training as a therapeutic approach for IR in children and adolescents with overweight and obesity, in order to inform policymakers, investors, and health professionals regarding the impact of exercise training on fasting glucose, fasting insulin, HOMA-IR, and body weight (BW).

## 2 Methods

### 2.1 Trial registration

This systematic review and meta-analysis was conducted according to the Preferred Reporting Items for Systematic Reviews and Meta Analyses (PRISMA) guidelines (11) and the Cochrane Handbook of Systematic Reviews of Interventions. The systematic review and meta-analysis was registered prospectively at the International Prospective Register of Systematic Reviews (PROSPERO) with the identification code: CRD42023391385.

### 2.2 Search strategy

A comprehensive electronic database search was completed in Scopus, Web of Science, and PubMed. Two reviewers (FK and FSH) independently identified published articles up to October 2022. Articles were searched using the keywords “exercise”, “training”, “physical activity”, “exercise training”, “sport”, “strength training”, “weight training”, “resistance training”, “progressive training”, “progressive resistance”, “weightlifting”, “aerobic exercise”, “aerobic training”, “endurance exercise”, “endurance training”, “cardio training”, “physical endurance”, and “physical exertion”. For identification of studies that assessed insulin resistance, search terms included “insulin”, “fasting insulin”, “glucose”, “fasting glucose”, “insulin resistance”, “IR”, “HOMA-IR”, “homeostatic model assessment for insulin resistance”, “insulin sensitivity”, “tolerance test”, “oral glucose tolerance test”, “OGTT”, “GTT”, “insulin tolerance test”, and “ITT”. In addition, the search strategy for identification of studies on childhood obesity, search terms included “pediatric”, “child”, “children”, “child*”, “adolescent*”, “teenager”, “girl”, and “boy”. Reference lists of all included studies were searched to ensure that relevant studies were not missed. The search was limited to articles written in the English language and studies conducted in human subjects. Articles were not limited based on publication date.

### 2.3 Inclusion and exclusion criteria

**Figure 1:**
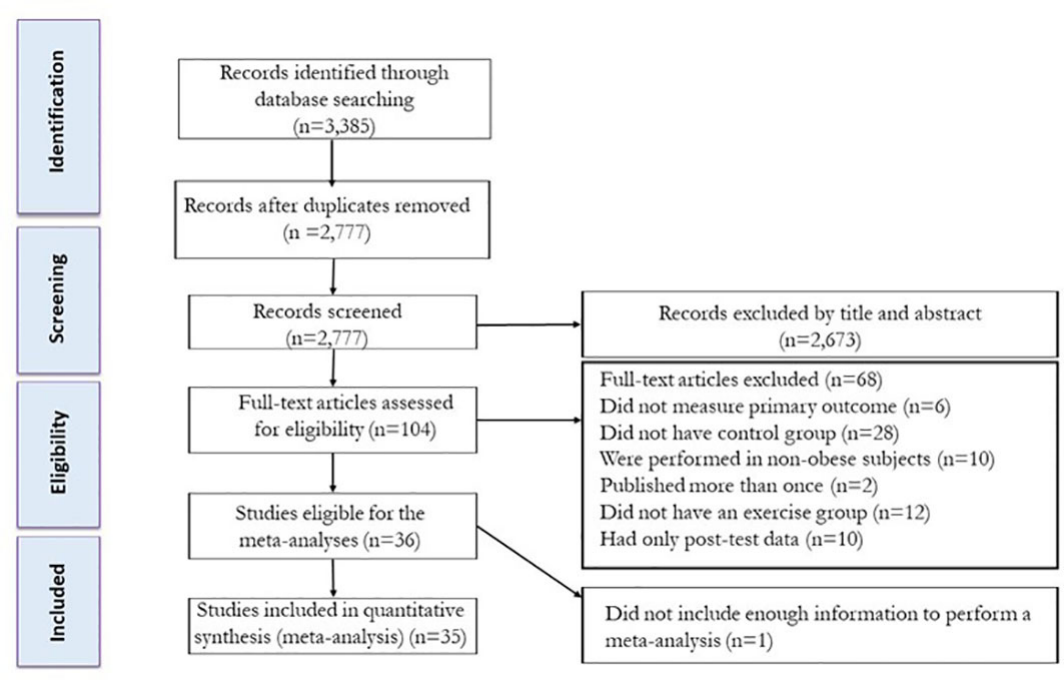
Flow diagram of systematic literature search.

**Figure 1** presents the flow of articles through the study selection process. Following removal of duplicate studies, titles and abstracts of articles were reviewed (initial screening) and then the full texts of potentially eligible studies were reviewed (secondary screening) by two independent reviewers (FK, and FSH) to determine eligibility for inclusion, and any disagreements were resolved by discussion with another author (NB). The following study characteristics were extracted: (A) participant characteristics including biological sex, age, body mass index (BMI), and sample size; (B) exercise protocol characteristics including exercise type, intensity, number of sessions per week, and intervention duration (weeks); (C) dietary intervention characteristics were extracted by the second author (FSH) with advice from FK on selection criteria. For each outcome (fasting insulin, fasting glucose, HOMA-IR, and body weight), the outcomes at pre- and post-intervention (means and standard deviations, or mean differences and associated standard deviations) were entered into the meta-analyses to generate forest plots. If the means and standard deviations (SDs) were not reported, the SDs were calculated from standard errors of means (SEM), or medians and interquartile ranges (IQRs) or means and IQRs (12–14).

Studies were eligible for inclusion when they met the following criteria (1): involved human subjects; (2) compared exercise *vs*. control; (3) measured at least one insulin resistance marker including fasting glucose, fasting insulin, HOMA-IR, or BW; (4) studied children or adolescents with overweight or obesity; (5) involved an intervention of at least 4 weeks in duration. Exclusion criteria were as follows: (1) non-original and non-experimental research such as case-control studies, study protocols, conference proceedings, letters to the editor, reviews, and meta-analyses; (2) animal studies; (3) studies in a non-English language; (4) studies where the diets of the exercise group and the control group were different; and (5) studies where participants had type 1 diabetes mellitus.

### 2.4 Quality evaluation and sensitivity analyses

Risk of bias was assessed using the Physiotherapy Evidence Database (PEDro) scale. We excluded two items (for no blinding of participants and intervention) from the original 11-item scale, because participants and intervention providers could not be blinded to the assigned exercise conditions during studies. The modified scale consisted of nine items: (1) specified eligibility (inclusion, and exclusion) criteria, (2) randomized participant allocation, (3) concealed allocation, (4) similarity of groups at baseline, (5) blinding of all assessors, (6) evaluated outcomes in 85% of participants, (7) intention-to-treat (ITT) analysis, (8) reporting of statistical comparisons between groups, (9) and point measures and statistics of variability (**Supplementary Table 1**).

### 2.5 Statistical analyses

Meta-analyses were implemented using Comprehensive Meta-analysis (CMA) software (version 2.0, Biostat Inc., NJ, USA) to calculate standardized mean differences (SMD) for fasting insulin, or weighted mean differences (WMD) for fasting glucose, HOMA-IR, body weight, and 95% confidence intervals (CIs) for outcomes using random-effects models. Because the units of measurement for fasting insulin were different across included studies, SMD were used. For other outcomes, where the units of measurement were the same across studies, WMD was used. These outcomes included fasting glucose (mg/dL), HOMA-IR, and body weight (kg). Effect sizes were calculated to compare the effects of exercise versus control groups (unexercised participants) on insulin resistance markers (fasting insulin, fasting glucose, HOMA-IR, or body weight). Heterogeneity was assessed using the I^2^ statistic. Significance was set at p<0.05. and evaluation of heterogeneity was conducted according to Cochrane guidelines as follows: 25% as low, 50% as moderate, and 75% as high heterogeneity. Subgroup analyses were performed according to the type of exercise (aerobic, resistance, or combined exercise), training duration (long-term interventions ≥8 weeks or shot-term interventions <8 weeks), biological sex (females, males, or females and males combined), BMI percentile (≥ 85th percentile as overweight, or ≥ 95th percentile as obesity), and health status (with or without diagnosed condition). In the included studies, diagnosed conditions included prehypertension, hyperinsulinemia, intellectual disability, or hypothyroidism. Publication bias was detected through funnel plot interpretation. If publication bias was present, Egger’s tests were used as a confirmatory test. Significant publication bias was deemed apparent if p<0.1 (15). Sensitivity analyses were performed by omitting individual studies to determine whether results were dependent on a single study.

## 3 Results

### 3.1 Included studies

Our initial search strategy identified 823 articles from Scopus, 707 articles from Web of Science, and 1,855 articles from PubMed. After eliminating duplicate records and screening titles and abstracts (initial screening), 104 studies were retrieved for a more detailed appraisal of the full texts (secondary screening). Sixty-eight studies were excluded after reviewing full texts for the following reasons: (A) six did not measure main outcomes (fasting glucose, fasting insulin, HOMA-IR, or body weight), (B) twenty-eight did not have a control group, (C) ten were performed in children or adolescents without overweight or obesity, (D) two were published more than once, (D) twelve did not include an exercise group, and (E) ten had only post-test data. In addition, one study did not have enough information to include in the meta-analysis. Corresponding authors were contacted; however, none provided the necessary information for these studies to be included. A total of 35 studies, inclusive of 45 intervention groups, were included in the present systematic review and meta-analysis. A flow diagram of the systematic literature search is presented in **Figure 1**.

### 3.2 Participant characteristics

A total of 1,550 children and adolescents were included with sample sizes ranging from 20 to 107. The mean age of participants ranged from 9 (16) to 18 years (17–19) and the mean BMIs of participants ranged from 21 (20) to 39 kg/m^2^ (21). The mean age of exercised participants was 14.5 ± 1.6 years, and the mean age of control groups was 14.1 ± 1.9 years. The mean BMI of exercised participants was 30.49 ± 3.73 kg/m^2^, and the mean BMI of control groups was 30.46 ± 4.03 kg/m^2^. Both males and females were included in eleven studies (16, 17, 19–27), females only in fifteen studies (28–41), and males only in ten studies (18, 42–50). All children and adolescents had overweight or obesity, one study included participants with hypothyroidism (30), and two studies included participants with prehypertension (28, 32). **Table 1** presents the full details of the participant characteristics.

**Table 1:** Characteristics of participants and exercise protocols.

Source, year	Study characteristics				Participant characteristics			Exercise characteristics			Dietary intervention characteristics
	Sample size (sex)	Groups	Intervention duration (~ week)	Outcomes	Health status	Age (years)	BMI (kg/m2)	Type	Protocol	Sessions/week	
Seo et al., 2012 (42)	20 M	Exe Con	8	FBG FI HOMA-IR BW	Obese	Exe: 14.7 ± 1.51 Con: 14.6 ± 3.03	Exe: 28.57 ± 3.88 Con: 29.04 ± 6.66	Yoga-asana	60 min at 50–60% HHR	3	Consuming a balanced diet
Chae et al., 2010 (22)	38 (M & F)	Exe Con	12	FBG FI HOMA-IR BW	Obese	Exe: 10.4 ± 13.48 Con: 10.6 ± 16.53	Exe: 26.6 ± 3.48 Con: 26.2 ± 4.35	Combined (Aerobic & Resistance	90 min	2	Instructed not to consume more than 1800–2000 kcal/day during weekdays. The control group with obesity underwent conventional counseling at the outpatient clinic.
de Lira et al., 2017 (43)	107 M	Exe1 Exe2 Con	12	FBG FI HOMA-IR	Obese	Exe1: 14.95 ± 1.35 Exe2: 14.77 ± 0.94 Con: 14.72 ± 1.35	Exe1: 35.36 ± 4.78 Exe2: 34.07 ± 4.05 Con: 35.09 ± 4.11	HIIT LIT	HIIT: 38 min at VT1 LIT: 52 min exercise at 20% below VT1	3	Participants were encouraged to reduce overall calorie intake and to follow a balanced diet.
Vissers et al., 2008 (17)	76 (M & F)	Exe Con	24	FBG	Overweight	Exe (F): 17.5 ± 1.3 Exe (M): 18.1 ± 1.3 Con (F): 17.1 ± 1.1 Con (M): 17.5 ± 1.4	Exe (F): 29.3 ± 2.9 Exe (M): 28.9 ± 2.0 Con (F): 29.3 ± 4.1 Con (M): 28.7 ± 3.6	Combined (Aerobic & Resistance	ND	3	Counseling by a dietitian
Alizadeh et al., 2019 (18)	20 M	Exe Con	6	FBG FI HOMA-IR BW	Overweight	Exe: 18.0 ± 1.5 Con: 18.0 ± 1.5	Exe: 27.8 ± 0.6 Con: 28.5 ± 0.6	SIT	14–16 min of 4–6 repetitions of 30s SIT with 30s recovery between each SIT with 90% MHR	3	control group participants were not allowed to take part in any exercise training and followed their routine daily activities and usual diets.
Rasooli et al., 2021 (44)	40 M	Exe Con	8	FBG FI HOMA-IR BW	Obese	Exe: 14-17 Con: 14-17	Exe: 33.8 ± 3.9 Con: 32.8 ± 3.8	Circuit resistance training	40–60 min of 2–4 sets of 6–12 repetitions at 70-80% 1RM	3	ND
Kim et al., 2020 (28)	48 F	Exe Con	12	FBG FI HOMA-IR BW	Prehypertension/Hyperinsulinemia/Overweight	Exe: 15 ± 1 Con: 15 ± 1	Exe: 28 ± 1 Con: 29 ± 1	Jump rope	50 min at 40–70% at 11–16 RPE	5	Participants were advised to maintain their current dietary habits.
Wong et al., 2018 (29)	30 F	Exe Con	12	FBG FI HOMA-IR BW	Hyperinsulinemia/Obese	Exe: 15.2 ± 1.2 Con: 15.3 ± 1.1	Exe: 30.1 ± 2.2 Con: 30.2 ± 1.2	Combined (Aerobic & Resistance)	T: 60 min A: 40–70% HRR R: 16–20 repetitions at 70–80% of maximum strength	3	Participants were reminded to make no changes to their diet.
Benson et al., 2008 (20)	78 (M & F)	Exe Con	8	FBG FI HOMA-IR	Overweight	Exe: 12.3 ± 1.3 Con: 12.2 ± 1.3	Exe: 23.2 ± 5.4 Con: 21.9 ± 3.6	Resistance	2 sets of 8 repetitions at 80% of peak strength (15–18 RPE scale)	2	ND
Sun et al., 2011 (23)	42 (M & F)	Exe Con	10	FBG FI HOMA-IR BW	Overweight	Exe (F): 13.8 ± 0.6 Exe (M): 13.4 ± 0.4 Con: 13.6 ± 0.7	Exe (F):26.9 ± 3 Exe (M): 27.1 ± 3.1 Con: 27.9 ± 3.8	Aerobic	60 min at 40–60% Vo_2max_	4	ND
Boer et al., 2014 (19)	54 (M & F)	Exe1 Exe2 Con	15	FBG FI HOMA-IR BW	Intellectual disability Overweight	Exe1: 18 ± 3.2 Exe2: 16.7 ± 3.6 Con: 17.4 ± 2.4	Exe1: 28.4 ± 4.7 Exe2: 27.5 ± 2.7 Con: 26.9 ± 3.2	SIT CAT	SIT: 40 min of 10×15s at >100 r/min at VTR with 45s rest at 50 r/min at VTR CAT: 50 min at 60–110% VTR r/min	2	ND
Sefat et al., 2019 (30)	20 F	Exe Con	8	FBG FI HOMA-IR BW	Hypothyroidism Overweight	Exe: 12.40 ± 1.71 Con: 11.80 ± 2.20	Exe: 26.02 ± 2.2 Con: 24.49 ± 3.3	Combined (Aerobic & Resistance)	T: 75 min A: 60–80% HRR R: 8–12 repetitions at 40-65% 1RM with 30s Rest	ND	ND
Meng et al., 2022 (45)	45 M	Exe1 Exe2 Con	12	FBG FI HOMA-IR	Obese	Exe1: 11.4 ± 0.8 Exe2: 11.2 ± 0.7 Con: 11.0 ± 0.7	Exe1: 24.5 ± 1.1 Exe2: 24.4 ± 0.9 Con: 23.8 ± 0.8	HIIT MICT	HIIT: 30–40 min 2 sets of eight bouts of 15s run at 90–100% MAS with eight bouts of 15s recovery runs between bouts at 50% MAS MICT: 30 min run with 60–70% MAS	3	Participants were asked to maintain their current diet throughout the duration of the study.
Lee et al., 2010 (46)	45 M	Exe1 Exe2 Con	12	FBG FI BW	Obese	Exe1: 15.2 ± 1.9 Exe2: 14.6 ± 1.5 Con: 14.8 ± 1.4	Exe1: 36.6 ± 5.9 Exe2: 34.5 ± 2.4 Con: 33.9 ± 4.2	Aerobic Resistance	A: 40–60 min at 50–75% of Vo_2peak_ R: 60 min of 8–12 repetitions at 60% 1RM with 1–2 min rest between sets	3	No caloric restriction
Dias et al., 2018 (24)	99 (M & F)	Exe1 Exe2 Con	12	FBG HOMA-IR	Obese	Exe1: 12.4 ± 1.9 Exe2: 11.9 ± 2.4 Con: 11.8 ± 2.4	Exe1: 28.8 ± 3.8 Exe2: 30.4 ± 5.0 Con: 29.6 ± 4.3	HIIT MICT	HIIT: 40 min 4 sets of 4 min bouts at 85–95% MHR with 3 min of recovery between bouts at 50–70% MHR MICT: 44 min at 60–70% MHR	3	Dietary guidelines with a particular focus on healthy food choices, portion sizes, and regular meal times.
Plavsic et al., 2020 (31)	44 F	Exe Con	12	HOMA-IR BW	Obese	Exe: 16.2 ± 1.3 Con: 15.5 ± 1.5	Exe: 32.6 ± 2.7 Con: 33.2 ± 3.5	HIIT	T: 43 min 4 sets of 4 min intervals at 85–90% MHR with a 3 min recovery at 70% MHR	2	All participants were asked to consume (1500-1700 kcal/day).
Son et al., 2017 (32)	40 F	Exe Con	12	FBG FI HOMA-IR BW	Prehypertension/Obese	Exe: 15 ± 4.47 Con: 15 ± 4.47	Exe: 30.36± 3.08 Con: 30.31± 3.39	Combined (Aerobic & Resistance)	60 min at 40–70% HRR (RPE 11–16)	3	Asked to maintain their usual diet.
Davis et al., 2009 (33)	34 F	Exe1 Exe2 Con	16	FBG FI BW	Overweight	Exe1: 15.7 ± 1.2 Exe2: 14.8 ± 1 Con: 15.3 ± 1.1	Exe1: 32.8. ± 7.9 Exe2: 33.6 ± 6.9 Con: 34.6 ± 7.4	Resistance Combined (Aerobic & Resistance)	R: 30 min of 1–3 sets with 5–15 repetitions C: 30 min total cardiovascular activity	2	All intervention groups received the same nutrition education class.
Abassi et al., 2020 (34)	24 F	Exe1 Exe2 Con	12	FBG FI HOMA-IR BW	Overweight	Exe1: 16.1 ± 0.99 Exe2: 16.5 ± 1.07 Con: 16.9 ± 1.64	Exe1: 32.5 ± 2.52 Exe2: 33.6 ± 3.66 Con: 32.7 ± 4.71	HIIT MIIT	HIIT: 50 min 2 sets of 6–8 bouts of 30s run at 100–110% MAS with 30s recovery between bouts at 50% MAS MIIT: 50 min 2 sets of 6–8 bouts of 30s run at 70–80% MAS, with 30s recovery between bouts at 50% MAS	3	ND
Zehsaz et al., 2016 (47)	32 M	Exe Con	16	FBG FI HOMA-IR BW	Obese	Exe: 10.8 ± 0.9 Con: 10.3 ± 0.9	Exe: 31.7 ± 1 Con: 31.7 ± 1	Combined (Aerobic & Resistance)	T: 85–90 min A: 30–35 min at 5575% MHR R: 55 min with 20 repetitions with 70% 1RM	4	Asked not to modify their diet during the study period.
Kelly et al., 2004 (25)	25 (M & F)	Exe Con	8	FBG FI BW	Overweight	Exe: 11 ± 1.99 Con: 11 ± 2.24	Exe: 32.1 ± 7.58 Con: 30.5 ± 7.26	Aerobic	T: 30 min at 50–80% Vo_2peak_	4	ND
Kelly et al., 2019 (48)	32 M	Exe Con	16	FBG	Obese	Exe: 15.29 ± 0.95 Con: 15.58 ± 0.99	Exe: 33.13 ± 4.30 Con: 34.22 ± 7.55	Resistance	R: 60 min of 1–4 sets of 3–16 repetitions with 62–97% 1RM	2	ND
Liu et al., 2018 (35)	50 F	Exe Con	4	FBG FI HOMA-IR BW	Obese	Exe: 14.6 ± 0.7 Con: 14.7 ± 0.8	Exe: 33.9 ± 3.1 Con: 34.6 ± 4.9	Aerobic	120 min with 5 min rest per 30 min (heart rate at the at 100–140 beats/min)	6 Twice a day	Total daily energy of 1400 or 1600 kcal (different in portion size, with carbohydrate, protein, and fat accounting for 65%, 15%, and 20% of the total energy, respectively.
Karacabey et al., 2009 (49)	40 M	Exe Con	12	FI BW	Obese	Exe: 11.8 ± 0.5 Con: 11.2 ± 0.8	Exe: 34.9 ± 4.1 Con: 35.5 ± 3.2	Aerobic (Walking-Jogging)	30–55 min at 60–65%HRR	3	All study participants undertook the same diet program.
Davis et al., 2011 (36)	31 F	Exe Con	16	FI HOMA-IR	Overweight	Exe: 15.7 ± 1.1 Con: 15.8 ± 1.0	Exe:32.4 ± 3.2 Con:36.4 ± 5.2	Combined (Aerobic & Resistance)	T: 60–90 min A: 70–85% MHR R: 8–14 repetitions of 6–9 circuits repeated twice: each consisting of two ST exercises (1 min each) and one cardiovascular exercise (2–3 min)	2	ND
Lee et al., 2013 (37)	44 F	Exe1 Exe2 Con	12	FBG FI BW	Obese	Exe1: 14.6 ± 1.9 Exe2: 14.8 ± 1.9 Con: 15.0 ± 2.2	Exe1: 32.9 ± 3.8 Exe2: 36.4 ± 3.8 Con: 35.3 ± 4.0	Aerobic Resistance	A: 40–60 min at 50–75%Vo_2_peak R: 60 min of 1–2 sets of 8–12 repetitions at 60% 1RM	3	All participants were asked to follow a weight maintenance diet.
McCormack et al., 2014 (21)	18 (M & F)	Exe Con	8	FBG FI HOMA-IR BW	Mild insulin resistance/Obese	Exe:13.8 ± 2.2 Con:12.1 ± 1.2	Exe: 39.1 ± 6.5 Con: 34.3 ± 7.4	Aerobic	20 min at 60–80% HRR	3	ND
Farpour-Lambert et al., 2009 (16)	44 (M & F)	Exe Con	12	FBG FI HOMA-IR BW	Pre-pubertal Obese	Exe: 9.1 ± 1.4 Con: 8.8 ± 1.6	Exe: 25.4 ± 4.6 Con: 25.1 ± 4.7	Combined (Aerobic & Resistance)	T: 60 min A: 55–65% Vo_2max_ R: 2–3 sets of 10–15 repetitions	3	No dietary intervention
Murphy et al., 2009 (26)	35 (M & F)	Exe Con	12	FBG FI HOMA-IR BW	Impaired FMD/Overweight	Exe: 10.21 ± 1.67 Con: 10.21 ± 1.67	Exe: 27.9 ± 4.8 Con: 31.8 ± 5	Dance	T: 10–30 min	5	ND
Meyer et al., 2006 (27)	96 (M & F)	Exe Con	24	FI HOMA-IR	Obese	Exe: 14.7 ± 2.2 Con: 14.7 ± 2.2	Exe: 29.8 ± 5.93 Con: 31 ± 4.42	Swimming and Aqua aerobic training/Sports games/Walking	T: 60 min	3	There was no change in calorie intake or diet plan in both groups.
Kim et al., 2011 (51)	30 M	Exe Con	12	FBG FI HOMA-IR BW	Overweight	Exe: 17.63 ± 0.49 Con: ND	Exe: 28.79 ± 3.81 Con: 28.33 ± 2.84	Aerobic	T: 50 min	5	ND
Lopes et al., 2016 (38)	40 F	Exe Con	12	FBG FI HOMA-IR BW	Overweight	Exe: 14.6 ± 1.15 Con: 14.4 ± 1.16	Exe: 28.8 ± 3.62 Con: 29.04 ± 3.09	Combined (Aerobic & Resistance)	T: 60 min A: 50–85% Vo_2peak_ R: 3 sets of 6–10 repetitions with 60–70% 1 RM	3	All participants were instructed to maintain their usual food intake throughout the study period.
Racil et al., 2013 (39)	34 F	Exe1 Exe2 Con	12	FBG FI HOMA-IR BW	Obese	Exe1: 15.6 ± 0.7 Exe2: 16.3 ± 0.52 Con: 15.9 ± 1.2	Exe1: BMI >97% centiles Exe2: BMI >97% centiles Con: BMI >97% centiles	HIIT MIIT	HIIT: 35–37 min 2 sets of 6–8 repetitions of 30s run at 100–110% of MAS with recovery bouts at 50% MAS MIIT: 35–37 2 sets 6–8 repetitions of 30s run with 70–80% of MAS with recovery between bouts at 50% MAS	3	Maintained usual diet
Racil et al., 2016 (41)	75 F	Exe1 Exe2 Con	12	FBG FI HOMA-IR BW	Obese	Exe1: 16.6 ± 0.9 Exe2: 16.5 ± 1.2 Con: 16.9 ± 1.0	Exe1(Z-score): 2.9 ± 0.2 Exe2 (Z-score): 2.9 ± 0.3 Con (Z-score): 2.8 ± 0.3	HIIT Combined (Plyometric exercise & HIIT)	HIIT: 44 min 2 sets of 6 repetitions or 8 bouts of 30s runs at 100% Vo_2peak_ with 30s recovery between bouts at 50% Vo_2peak_ P+HIIT: 50 min two blocks of three different plyometric exercises with HIIT at 100% Vo_2peak_	3	Maintained usual diet
Vasconcellos et al., 2015 (40)	20 F	Exe Con	12	FBG FI HOMA-IR BW	Obese	Exe: 14.1 ± 1.3 Con: 14.8 ± 1.4	Exe: 31.1 ± 5.2 Con: 32.2 ± 4.9	Soccer	T: 60 min	3	ND

M, Male; F, Female; G, Girl; B, Boy; Exe, Exercise; Con, Control; DI, diet intervention; FI, Fasting insulin; FBG, Fasting blood glucose; HOMA-IR, Homeostatic Model Assessment for Insulin Resistance; ND, Not-described; HIIT, High-intensity interval training; MICT, Moderate-intensity continuous training; MIIT, Moderate-intensity interval training; LIT, Low-intensity training; MAS, Maximal aerobic speed; VT_R_, Ventilatory threshold; 1RM, One-repetition maximum; HRR, Heart rate reserve; MHR, Maximal heart rate; A, Aerobic; R, Resistance; VO_2_peak/max, Peak or maximal oxygen uptake; VT1, Ventilatory threshold 1; SIT, Sprint interval trainig; CAT, Continuous aerobic training; S, Seconds; RPE, Rating of perceived exertion;T, Time of each training session.

### 3.3 Intervention characteristics

Intervention durations ranged from 4 (35) to 24 weeks (17, 27), with 12-week durations in the majority of studies. In 21 studies aerobic exercise was compared with a control (18, 19, 21, 23–29, 31, 34, 35, 39–41, 43, 45, 46, 49, 50, 52), 6 studies compared resistance exercise vs. control (20, 33, 44, 46, 48, 52), and 11 studies compared combined exercise vs. control (16, 17, 22, 29, 30, 32, 33, 36, 38, 41, 47), while one study used yoga vs. control (42). Ten studies used more than one type of exercise protocol as separate interventions (19, 24, 33, 34, 37, 39, 41, 43, 45, 46). Exercise training sessions were performed 2–5 times per week, with 3 sessions being the most common (n = 20) (16–18, 21, 24, 27, 29, 32, 34, 37–46, 49). In one study, exercise was performed twice a day (35). The duration of each session of aerobic training ranged from 10–15 min (18, 26) to 120 min (35). The duration of each session of resistance training ranged from 30 min (33) to 60 min (44, 46, 48, 52), and one study did not mention session duration (20). The duration of each session of combined exercise varied from 30 min (33), to 50 min, to 90 min (16, 22, 29, 30, 32, 36, 38, 41, 47), and one study did not mention session duration (17). The intervention characteristics are detailed in **Table 1**.

### 3.4 Meta-analysis

#### 3.4.1 Exercise training vs. control

##### 3.4.1.1 Fasting glucose

**Figure 2:**
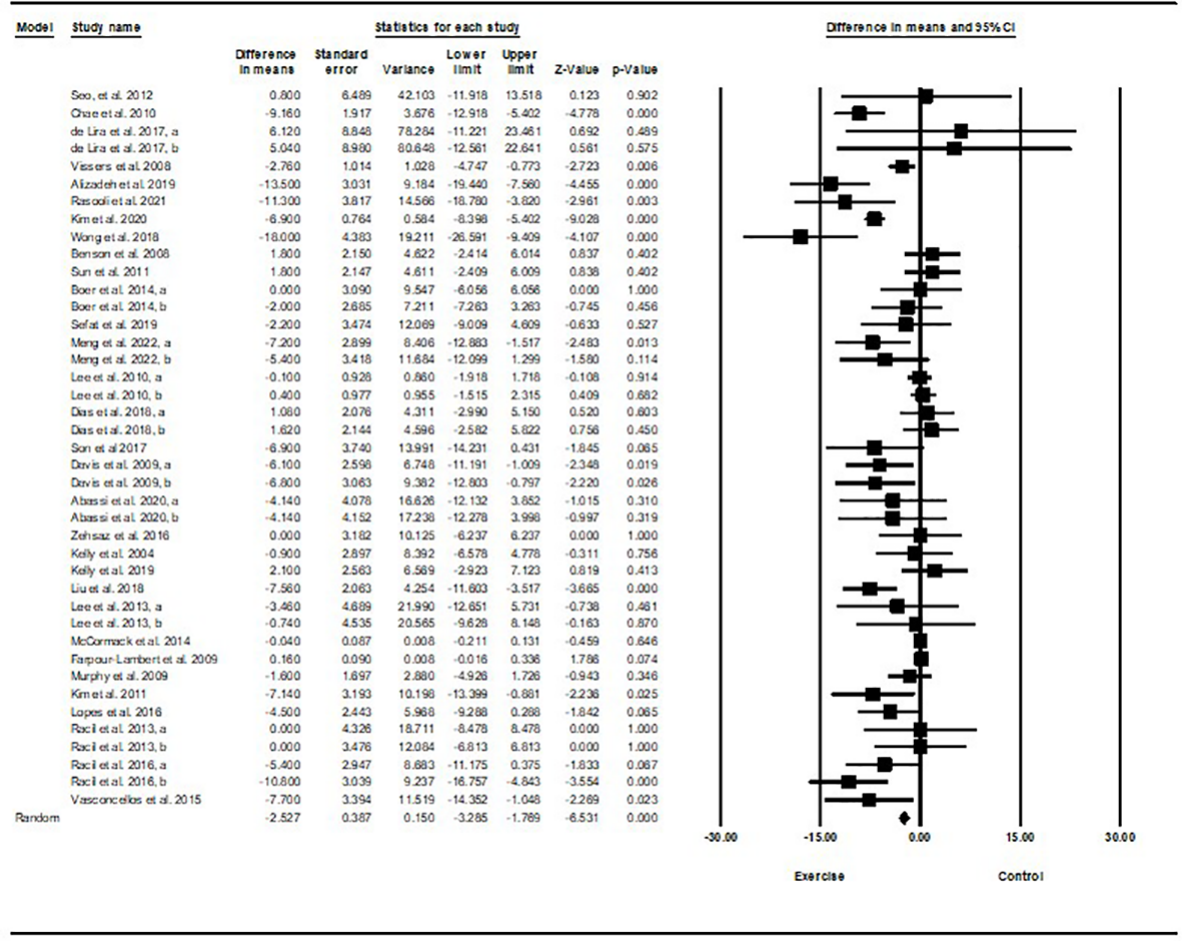
Forest plot of the effects of exercise training vs. control on fasting glucose. Data are reported as WMD (mg/dL) (95% confidence limits). WMD, weighted mean difference.

Based on 41 intervention arms with 1,256 participants, exercise training effectively reduced fasting glucose [WMD=-2.52 mg/dL (95% CI -3.28 to -1.76), p=0.001], when compared to control groups (**Figure 2**).

Subgroup analyses revealed significant reductions in fasting glucose for aerobic exercise [WMD=-2.85 mg/dL (95% CI -4.59 to -1.10), p=0.001, 25 interventions], combined exercise [WMD=-5.43 mg/dL (95% CI -8.48 to -2.38), p=0.001, 10 interventions], but not for resistance training [WMD=-1.63 mg/dL (95% CI -5.02 to 1.77), p=0.34, 6 interventions], compared with a control group. In addition, subgroup analyses revealed significant reductions in fasting glucose for long-term interventions ≥8 weeks [WMD=-3.12 mg/dL (95% CI -4.57 to -1.68), p=0.001, 33 interventions], as well as short-term intervention <8 weeks [WMD=-4.03 mg/dL (95% CI -7.83 to -0.24), p=0.03, 8 interventions] compared with a control group. Subgroup analyses by sex indicated a significant reduction in fasting glucose for females [WMD=-6.06 mg/dL (95% CI -7.58 to -4.53), p=0.001, 17 interventions], and males [WMD=-3.31 mg/dL (95% CI -6.14 to -0.47), p=0.02, 12 interventions], but not for females and males combined [WMD=-0.29 mg/dL (95% CI -0.84 to 0.26), p=0.3, 12 interventions], compared with a control group. In addition, subgroup analyses by BMI percentile indicated a significant reduction in fasting glucose for adolescents with overweight [WMD=-3.54 mg/dL (95% CI -5.47 to -1.62), p=0.001, 16 interventions], and with obesity [WMD=-1.2 mg/dL (95% CI -1.96 to -0.45), p=0.002, 25 interventions], compared with a control group. Subgroup analyses by health status indicated a significant reduction in fasting glucose for adolescents without another diagnosed health condition [WMD=-3.04 mg/dL (95% CI -4.4 to -1.69), p=0.001, 3 interventions], and adolescents with another diagnosed condition [WMD=-4.03 mg/dL (95% CI -7.54 to -0.51), p=0.02, 8 interventions], compared with a control group (**Supplementary Table 2**).

There was significant heterogeneity among included studies (I^2 = ^82.88%, p=0.001). Visual interpretation of funnel plots and Egger’s test (p=0.001) results also showed publication bias. Sensitivity analysis performed by removing individual studies showed that significance of results and direction of the results did not change.

##### 3.4.1.2 Fasting insulin

**Figure 3:**
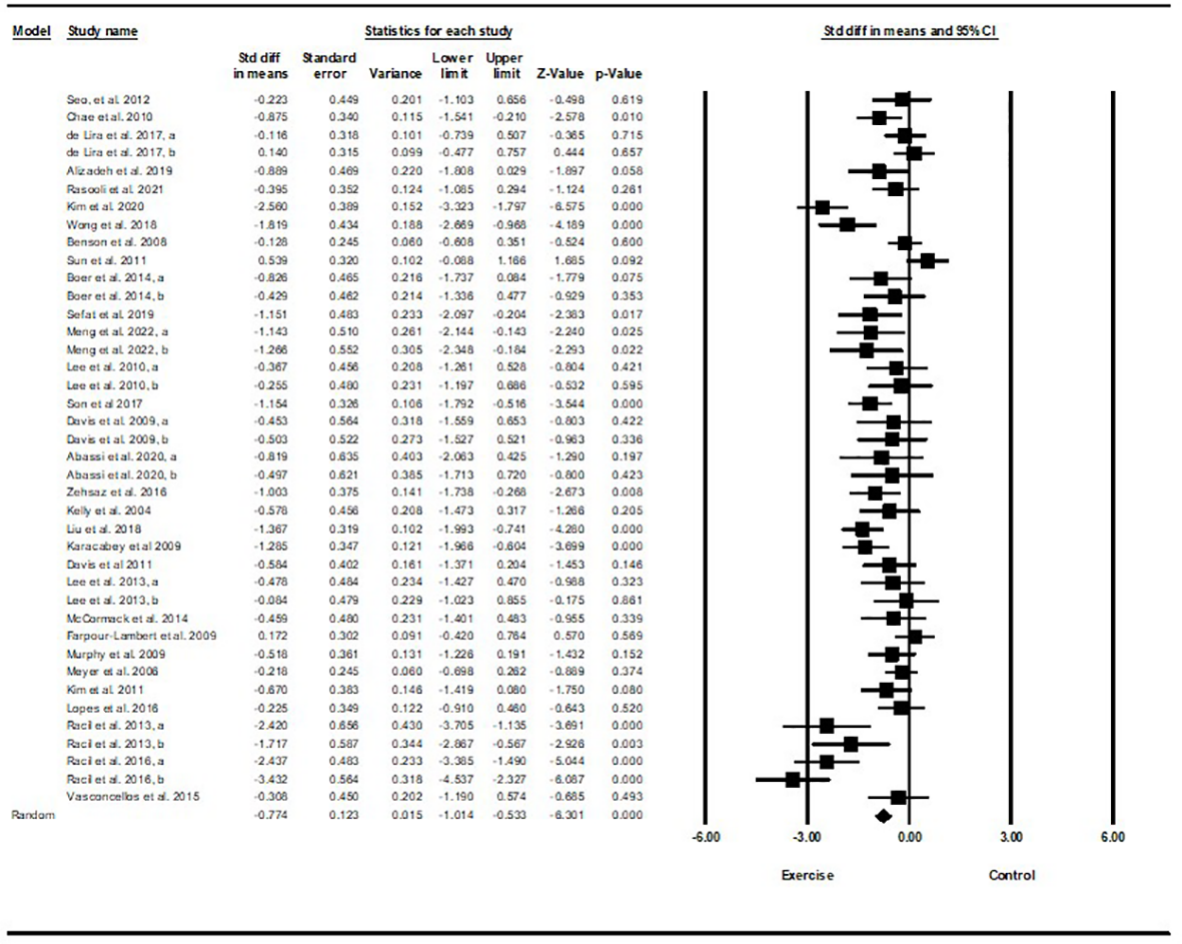
Forest plot of the effects of exercise training vs. control on fasting insulin. Data are reported as SMD (95% confidence limits). SMD, standardized mean difference.

Based on 40 intervention arms with 1,192 participants, exercise training effectively reduced fasting insulin [SMD=-0.77 (95% CI -1.01 to -0.53), p=0.001], when compared with a control group, with a medium effect size (-0.77) (**Figure 3**).

Subgroup analyses by type of exercise revealed a significant reduction in fasting insulin for aerobic exercise [SMD=-0.79 (95% CI –1.1 to -0.47), p=0.001, 25 interventions], and combined exercise [SMD=-1.0 (95% CI -1.53 to -0.46), p=0.001, 10 interventions], but not for resistance training [SMD=-0.22 (95% CI -0.54 to 0.09), p=0.17, 5 interventions], compared with a control group. In addition, subgroup analyses by intervention duration indicated a significant reduction in fasting insulin for intervention durations of more than 8 weeks [SMD=-0.81 (95% CI -1.1 to -0.52), p=0.001, 32 interventions], as well as durations of less than 8 weeks [SMD=-0.62 (95% CI -0.98 to -0.26), p=0.001, 8 interventions] compared with a control group. In addition, subgroup analyses by biological sex indicated a significant reduction in fasting insulin for females [SMD=-1.2 (95% CI -1.63 to -0.78), p=0.001, 18 interventions], and males [SMD=-0.57 (95% CI -0.86 to -0.28), p=0.001, 12 interventions], but not for females and males combined [SMD=-0.26 (95% CI -0.54 to 0.007), p=0.05, 10 interventions], compared with a control group. In addition, subgroup analyses by BMI percentile indicated a significant reduction in fasting insulin for adolescents with overweight [SMD=-0.62 (95% CI -0.97 to -0.26), p=0.001, 16 interventions], and with obesity [SMD=-0.87 (95% CI -1.2 to -0.55), p=0.001, 24 interventions], compared with a control group. Subgroup analyses by health status indicated a significant reduction in fasting insulin for adolescents without another diagnosed condition [SMD=-0.67 (95% CI -0.93 to -0.42), p=0.001, 32 interventions], and adolescents with another diagnosed condition [SMD=-1.12 (95% CI -1.66 to -0.59), p=0.001, 8 interventions], compared with a control group (**Supplementary Table 2**).

There was significant heterogeneity among included studies (I^2 = ^72.27%, p=0.001). Visual interpretation of funnel plots and Egger’s test (p=0.005) results indicated publication bias. Sensitivity analysis performed by omitting each individual study showed that the effect size and significance of results and direction of the results did not change.

##### 3.4.1.3 HOMA-IR

**Figure 4:**
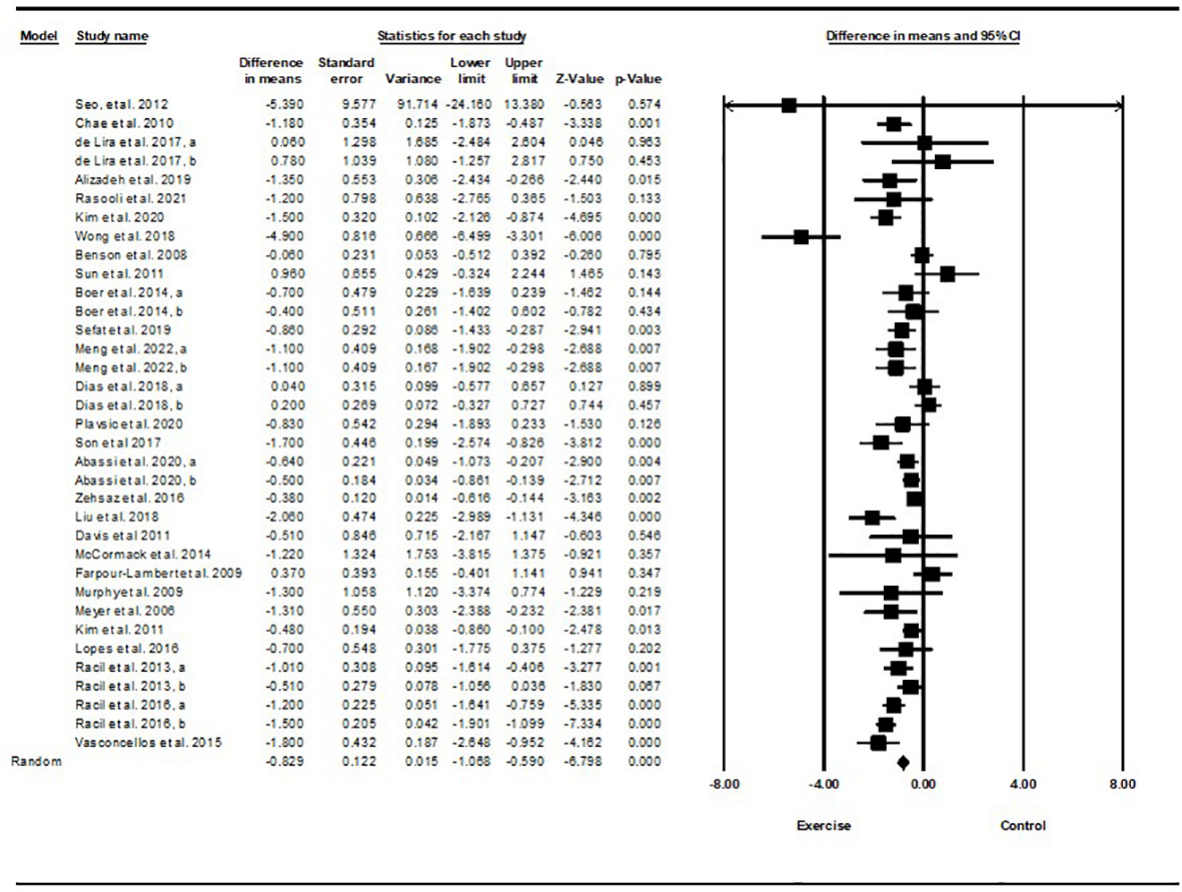
Forest plot of the effects of exercise training vs. control on HOMA-IR. Data are reported as WMD (95% confidence limits). WMD, weighted mean difference.

Based on 35 intervention arms with 1,152 participants, exercise training effectively reduced HOMA-IR [WMD=-0.82 (95% CI -1.07 to -0.59), p=0.001], when compared with a control group (**Figure 4**).

Subgroup analyses revealed significant reductions in HOMA-IR for aerobic exercise [WMD=-0.76 (95% CI -1.03 to -0.5), p=0.001, 23 interventions], combined exercise [WMD=-1.11 (95% CI -1.71 to -0.51), p=0.001, 9 interventions], but not for resistance training [WMD=-0.37 (95% CI -1.37 to 0.62), p=0.46, 2 interventions], compared with a control group. In addition, subgroup analyses revealed significant reductions in HOMA-IR for long-term interventions (≥8 weeks) [WMD=-0.79 (95% CI -1.06 to -0.53), p=0.001, 27 interventions], and short-term interventions (<8 weeks) [WMD=-1.03 (95% CI -1.72 to -0.33), p=0.004, 7 interventions] compared with a control group. In addition, subgroup analyses by biological sex indicated a significant reduction in HOMA-IR for females [WMD=-1.23 (95% CI -1.58 to -0.87), p=0.001, 14 interventions], males [WMD=-0.50 (95% CI -0.69 to -0.32), p=0.001, 9 interventions], but not for females and males combined [WMD=-0.27 (95% CI -0.66 to 0.11), p=0.16, 11 interventions], compared with a control group. In addition, subgroup analyses by BMI percentile indicated a significant reduction in HOMA-IR for adolescents with overweight [WMD=-0.6 (95% CI -0.87 to -0.34), p=0.001, 13 interventions], and with obesity [WMD=-0.99 (95% CI -1.36 to -0.62), p=0.001, 21 interventions], compared with a control group. Subgroup analyses by health status indicated a significant reduction in HOMA-IR for adolescents without another diagnosed condition [WMD=-0.69 (95% CI -0.93 to -0.44), p=0.001, 26 interventions], and those with another diagnosed condition [WMD=-1.45 (95% CI -2.16 to -0.74), p=0.001, 8 interventions], compared with a control group (**Supplementary Table 2**).

There was significant heterogeneity among included studies (I^2 = ^75.11%, p=0.001). Visual interpretation of funnel plots and Egger’s test (p=0.12) results did not show publication bias. Sensitivity analysis performed by removing individual studies revealed that significance of the results and direction of the results did not change.

##### 3.4.1.4 Body weight

**Figure 5:**
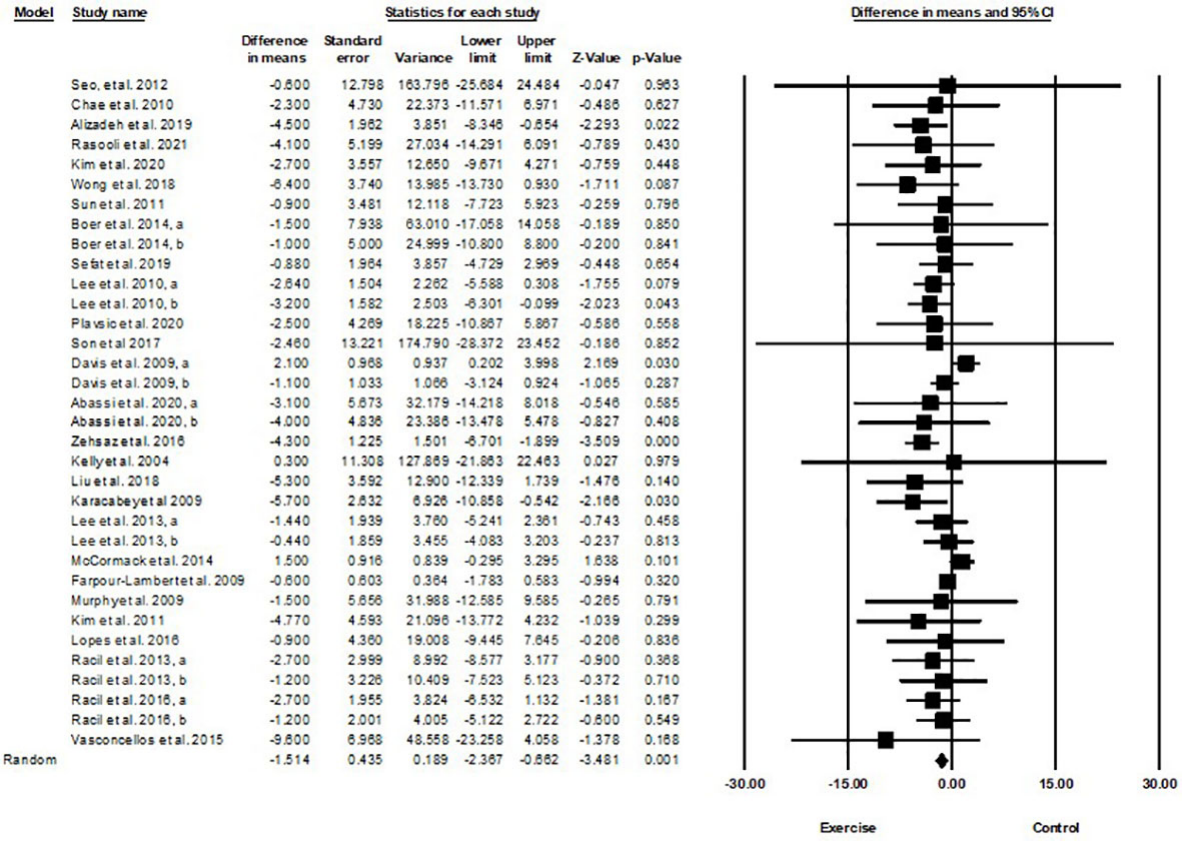
Forest plot of the effects of exercise training vs. control on body weight. Data are reported as WMD (kg) (95% confidence limits). WMD, weighted mean differences.

Based on 34 intervention arms with 949 participants, exercise training effectively reduced body weight [WMD=-1.51 kg (95% CI -2.36 to -0.66), p=0.001] in children and adolescents with overweight and obesity, when compared with a control group (**Figure 5**).

Subgroup analyses by type of exercise revealed a significant reduction in body weight for aerobic exercise [WMD=-1.44 kg (95% CI -2.54 to -0.34), p=0.01, 21 interventions], combined exercise [WMD=-1.55 kg (95% CI -2.66 to -0.44), p=0.006, 9 interventions], but not for resistance training [WMD=-0.61 kg (95% CI -3.74 to 2.5), p=0.69, 4 interventions], compared with a control group. In addition, subgroup analyses by intervention duration indicated a significant reduction in body weight for intervention durations of more than 8 weeks [WMD=-1.54 kg (95% CI -2.4 to -0.69), p=0.001, 27 interventions], but not for durations of less than 8 weeks [WMD=-1.55 kg (95% CI -4.33 to 1.21), p=0.27, 7 interventions] compared with a control group. In addition, subgroup analyses by sex indicated a significant reduction in body weight for males [WMD=-3.83 kg (95% CI -5.22 to -2.45), p=0.001, 8 interventions], but not for females [WMD=-0.67 kg (95% CI -1.66 to 0.3), p=0.17, 18 interventions], or females and males combined [WMD=-0.03 kg (95% CI -0.99 to 0.92), p=0.94, 8 interventions], compared with a control group. In addition, subgroup analyses by BMI percentile indicated a significant reduction in body weight for adolescents with obesity [WMD=-1.92 kg (95% CI -3.02 to -0.82), p=0.001, 20 interventions], but not for those with overweight [WMD=-0.38 kg (95% CI -1.52 to 0.76), p=0.51, 14 interventions], compared with a control group. Subgroup analyses by health status indicated a significant reduction in body weight for adolescents without another diagnosed condition [WMD=-1.35 kg (95% CI -2.02 to -0.67), p=0.001, 27 interventions], but not for those with another diagnosed condition [WMD=0.60 kg (95% CI -0.93 to 2.14), p=0.4, 7 interventions], compared with a control group (**Supplementary Table 2**).

There was not significant heterogeneity among included studies (I^2 = ^23.57%, p=0.1). Visual interpretation of funnel plots and Egger’s test (p=0.009) results indicated publication bias. Sensitivity analysis performed by omitting an individual study showed that significance and direction of the results did not change.

### 3.5 Quality assessment

The methodological quality of individual studies was evaluated using the PEDro tool with scores ranging from 3–9 out of a maximum of 9 points. One study had score of 9, eleven studies had scores of 6, four studies scored 7, fourteen scored 5, three studies scored 4, and two studies scored 3. Most of the studies received lower scores due to three evaluation criteria (concealed allocation, blinding of all assessors, and intention-to-treat analysis). The details of the quality of studies are provided in **Supplementary Table 1**.

## 4 Discussion

Childhood and adolescent obesity induces IR, which is a strong determinant of chronic diseases in adulthood (4). Childhood and adolescence are particularly vulnerable times for the development of obesity-induced IR due to metabolic and hormonal changes, which might be concomitant with a reduction in physical activity (5, 53). Interestingly, health intervention strategies undertaken during or prior to puberty may have greater positive effects on health outcomes than many interventions undertaken by adults (5, 53). Although complex multi-component interventions have been suggested to maximize impact, exercise is one of the most powerful strategies for improving obesity-induced IR (7).

The present meta-analysis, with 35 included studies, indicated that exercise training significantly decreases fasting glucose, insulin levels, HOMA-IR, and BW compared with a control group. Subgroup analyses indicated significant reductions in fasting glucose, insulin levels, and HOMA-IR with both aerobic and combined exercise interventions as compared to a control, but this did not hold true for resistance training. Exercise training was effective regardless of the length of the intervention (>8 and ≤8 weeks), the BMI percentiles (overweight/obesity), biological sex (female/male), and health status (with and without other diagnoses). A possible explanation for being effective the aerobic and combined exercise and not resistance training is that the physiologic adaptations to aerobic and resistance training are distinct. Aerobic training is characterized by the execution of cyclic exercises, carried out with large muscle groups, at mild to moderate intensities, for a long period of time with higher energy expenditure as compared to other types of exercise. On the other hand, resistance training (also called weight or strength training) is characterized by the execution of exercises in which muscles from a specific body part are contracted against a force that opposes the movement. So, it is more typical to design aerobic training interventions with a focus on metabolic changes/weight loss compared to resistance training, which is usually focused on musculoskeletal outcomes. In addition, it is well established that aerobic training decreases insulin resistance, and is associated with anti-inflammatory effects (10).

It is worth mentioning that subgroup analyses according to health status revealed significant reductions in fasting glucose, insulin levels, and HOMA-IR in children and adolescents with overweight and obesity (without other diagnoses), as compared with those who had other diagnoses, though fewer studies were included in this sub-group. It has been shown previously that participants with overweight or obesity who have comorbid conditions may benefit more from interventions, since they often have more metabolic dysregulation (54). However, there are many unknowns when it comes to understanding the optimal types and amounts of exercise in this population, and who would benefit the most from different types of interventions. It is unknown whether combined aerobic and resistance training is associated with greater improvements in outcomes than either type of exercise alone, or whether the responses would be similar in both biological sexes, across different lengths of interventions, and whether they would be dependent on overweight or obesity status or having other diagnosed health conditions. Further investigations should shed light on these factors to help determine the best recommendations for exercise training interventions.

Subgroup analyses for BW indicated significant reductions in BW with interventions that were longer than 8 weeks, which may be expected given the assumption that each MET-h/week is associated with a 0.33kg decrease in body weight (55). In addition, there were significant reductions in BW for adolescents with obesity, males, those without other diagnosed conditions, and for both aerobic and combined exercise interventions. Perhaps testosterone/skeletal muscle mass in adolescent boys may enhance some of the benefits of exercise on BW (10, 56).

A previously published meta-analysis assessed the associations between exercise training and changes in IR, fasting glucose, and fasting insulin in children and adolescents with overweight or obesity. The pooled mean effect size for the 17 included studies showed that exercise training, especially aerobic training, was significantly associated with reductions in fasting insulin levels (-3.37Uu/ml) and HOMA-IR (-0.61) (10). Very recently another meta-analysis reported that exercise in children/adolescents with overweight, effectively reduced blood glucose(0.58) and BMI (0.38) (57). The pooled mean effect size for the 26 included trials in another meta-analysis showed that exercise can significantly decrease BMI (-0.52) and HbA1c (-0.41) in children/adolescents with Type 1 diabetes (58). One other published meta-analysis reported significant reductions in BMI, fat mass, and percent body fat in children and adolescents with overweight and obesity when comparing exercise groups vs. control groups. Combined aerobic and strength training interventions were the most effective for improving both fat mass and percent body fat, while aerobic exercise was the most effective for improving BMI (59). However another meta-analysis showed no significant differences in HOMA-IR, blood glucose and body weight in a high-intensity interval training intervention compared with moderate ones in children and adolescents with obesity (9).

There are some inconsistent findings between the aforementioned meta-analyses. The meta-analyses were conducted at different points in time, and therefore included different studies. Recent studies could not be considered and different methods, and inclusion criteria, of studies used likely influenced the findings. The results described here also might be potentially due to other lifestyle factors, adiposity levels, race/ethnicity, pubertal status, biological sex, whether they were small for gestational age or premature (5), and differences in genetic factors such as single nucleotide polymorphisms (SNPs), which could modify the tissue responses to exercise (60).

The mechanisms by which exercise has favorable effects on indices related to overweight and obesity-induced IR, are diverse. As excess adipose tissue is associated with incident IR, exercise could improve indices of IR via decreases in adipose tissue, in particular, visceral tissue (61). Excess adipose tissue releases excess FFAs, resulting in lipotoxicity and dysregulated organelles, including endoplasmic reticulum stress and mitochondrial dysfunction (3, 4). Such organelles generate excess reactive oxygen species (ROS) and pro-inflammatory cytokines. These molecules interact with their receptors on cells, activating a series of signaling pathways, ultimately disrupting insulin signaling pathways and subsequently leading to IR (62). Additionally, excess adipose tissue may increase the expression and activity of xanthine oxidoreductase enzymes resulting in the production and secretion of uric acid. Increased serum uric acid plays a partial mediating role in obesity-induced IR in children and adolescents with obesity (63).

Aside from adipose tissue reduction, during exercise, particularly aerobic exercise, changes in metabolic and non-metabolic processes lead to improvement in IR and related indices. Exercise can reduce oxidative stress and inflammatory adipokines, and activate as well as increase the density of glucose transporter 4 (GLUT-4) (61, 64). Additionally, exercise is associated with the improvement of β-cell function, modulation of insulin receptor substrate 1 (IRS1) phosphorylation, lowering of ceramide plasma levels, and induction of angiogenesis, which are also mechanisms by which exercise improves overweight and obesity-induced IR (61, 64). Exercise also may induce weight loss by increasing daily energy expenditure, and potentially by suppressing appetite (65, 66).

Strengths of the current systematic review and meta-analysis include low publication bias, as well as subgroup analyses. In addition, we minimized potential bias in the review process by performing a comprehensive search of the literature and also by conducting the systematic review and reporting the meta-analytic results according to the PRISMA guidelines.

Despite the rigor of the present meta-analysis, some limitations should be considered before interpreting these results. As obesity-induced IR is a multi-factorial disorder with many related contributors, confounding variables that have not been controlled for within included studies, such as genetic background, daily functioning, environment, and other lifestyle factors may obscure the independent effects of exercise training. Moreover, clinical significance, referring to the practical or applied importance of outcomes, and whether they make a difference to patients in everyday life, is not clear for all outcomes.

### 4.1 Implications for practice

The current meta-analysis suggests that exercise training has beneficial effects on fasting glucose and insulin levels, HOMA-IR, and BW in children and adolescents with overweight or obesity. Though some of these effects are small, we should keep in mind that these outcomes were obtained with no additional requirements for caloric restriction. This is important since adherence to a restrictive diet may be challenging for children and adolescents with overweight or obesity. Additionally, overall lifestyle modification, not just exercise training, may be a first-line approach for the treatment of overweight and obesity. A dietary component for clinical management of IR and obesity would be an important addition to exercise training, particularly given the evidence for the efficacy of combined exercise and dietary interventions.

Current results cannot be generalized to those with other medical conditions and diseases as those populations were not included in this analysis.

### 4.2 Implications for research

Future research should include large, high-quality trials, with longer intervention durations and follow-up periods. These trials should be designed to ensure low risk of bias and to meet current reporting standards for clinical trials. With weight loss interventions, it is clear that weight maintenance is a challenge, and the same may be true of exercise interventions for long-term improvements in overweight and obesity-related IR and associated outcomes in children and adolescents.

## 5 Conclusion

Exercise training, particularly aerobic and combined exercise has beneficial effects on fasting insulin and glucose levels, HOMA-IR and BW in children and adolescents with overweight or obesity, and could provide an important adjunct therapy to control IR and related outcomes. It is essential to determine effective approaches for increasing exercise training in children and adolescents with overweight or obesity.

## Author contributions

FK, FS, and NB conceived and designed the study and extracted data. FK analyzed the data and completed the initial draft of the results. FK and MM drafted the initial manuscript. SR revised the manuscript. FK, MM, and SR read and approved the final manuscript. All authors contributed to the article.
