# Supplementary material for: The effects of exercise training on insulin resistance in children and adolescents with overweight or obesity: a systematic review and meta-analysis
Source: Front Endocrinol (Lausanne). 2023 Aug 10;14:1178376. doi: 10.3389/fendo.2023.1178376 (PMC10450243; doi:10.3389/fendo.2023.1178376)
Supplement: Supplementary file 1 [file Table_1.docx]

| **Supplementary Table 1.** Quality assessment of studies (PEDro Scale) | | | | | | | | | | |
| --- | --- | --- | --- | --- | --- | --- | --- | --- | --- | --- |
| Authors and Year of Publication | 1 | 2 | 3 | 4 | 5 | 6 | 7 | 8 | 9 | Total score |
| Seo, et al., 2012 (42) |  |  |  |  |  |  |  |  |  | 5 |
| Chae et al., 2010 (72) |  |  |  |  |  |  |  |  |  | 6 |
| de Lira et al., 2017 (73) |  |  |  |  |  |  |  |  |  | 6 |
| Vissers et al., 2008 (17) |  |  |  |  |  |  |  |  |  | 5 |
| Alizadeh et al., 2019 (18) |  |  |  |  |  |  |  |  |  | 4 |
| Rasooli et al., 2021 (74) |  |  |  |  |  |  |  |  |  | 4 |
| Kim et al., 2020 (51) |  |  |  |  |  |  |  |  |  | 5 |
| Wong et al., 2018 (29) |  |  |  |  |  |  |  |  |  | 6 |
| Benson et al., 2008 (20) |  |  |  |  |  |  |  |  |  | 9 |
| Sun et al., 2011 (23) |  |  |  |  |  |  |  |  |  | 5 |
| Boer et al., 2014 (19) |  |  |  |  |  |  |  |  |  | 5 |
| Sefat et al., 2019 (30) |  |  |  |  |  |  |  |  |  | 5 |
| Meng et al., 2022 (45) |  |  |  |  |  |  |  |  |  | 5 |
| Lee et al., 2010 (46) |  |  |  |  |  |  |  |  |  | 6 |
| Dias et al., 2018 (24) |  |  |  |  |  |  |  |  |  | 7 |
| Plavsic et al., 2020 (31) |  |  |  |  |  |  |  |  |  | 6 |
| Son et al., 2017 (32) |  |  |  |  |  |  |  |  |  | 6 |
| Davis et al., 2009 (33) |  |  |  |  |  |  |  |  |  | 5 |
| Abassi et al., 2020 (34) |  |  |  |  |  |  |  |  |  | 6 |
| Zehsaz et al., 2016 (47) |  |  |  |  |  |  |  |  |  | 6 |
| Kelly et al., 2004 (25) |  |  |  |  |  |  |  |  |  | 3 |
| Kelly et al., 2019 (48) |  |  |  |  |  |  |  |  |  | 7 |
| Liu et al., 2018 (35) |  |  |  |  |  |  |  |  |  | 3 |
| Karacabey et al., 2009 (49) |  |  |  |  |  |  |  |  |  | 7 |
| Davis et al., 2011 (36) |  |  |  |  |  |  |  |  |  | 6 |
| Lee et al., 2013 (37) |  |  |  |  |  |  |  |  |  | 5 |
| McCormack et al., 2014 (21) |  |  |  |  |  |  |  |  |  | 5 |
| Farpour-Lambert et al., 2009 (16) |  |  |  |  |  |  |  |  |  | 7 |
| Murphy et al., 2009 (26) |  |  |  |  |  |  |  |  |  | 5 |
| Meyer et al., 2006 (27) |  |  |  |  |  |  |  |  |  | 6 |
| Kim et al., 2011 (75) |  |  |  |  |  |  |  |  |  | 5 |
| Lopes et al., 2016 (38) |  |  |  |  |  |  |  |  |  | 4 |
| Racil et al., 2013 (39) |  |  |  |  |  |  |  |  |  | 5 |
| Racil et al., 2016 (41) |  |  |  |  |  |  |  |  |  | 5 |
| Vasconcellos et al., 2015 (40) |  |  |  |  |  |  |  |  |  | 6 |

(1) specified eligibility (inclusion, and exclusion) criteria, (2) randomized participant allocation, (3) concealed allocation, (4) similarity of groups at baseline, (5) blinding of all assessors, (6) evaluated outcomes in 85% of participants, (7) intention-to-treat (ITT) analysis, (8) reporting of statistical comparisons between groups, (9) and point measures and statistics of variability

**Supplementary Table 2:** Summary of subgroup analyses for the effects of exercise training on body weight and insulin resistance markers

|  | **Subgroups** | **Number of studies (n)** | **SMD or WMD (95% CI)** | **p-value** | **p-heterogeneity** |
| --- | --- | --- | --- | --- | --- |
| **Fasting glucose** | | | | | |
| Type of exercise | Aerobic | 25 | WMD=-2.85 mg/dL (95% CI -4.59 to -1.10) | 0.001 | 0.001 |
|  | Combined | 10 | WMD=-5.43 mg/dL (95% CI -8.48 to -2.38) | 0.001 | 0.001 |
|  | Resistance | 6 | WMD=-1.63 mg/dL (95% CI -5.02 to 1.77) | 0.34 | 0.008 |
| Intervention duration | long-term >8 week | 33 | WMD=-3.12 mg/dL (95% CI -4.57 to -1.68) | 0.001 | 0.001 |
|  | Short-term ≤8 week | 8 | WMD=-4.03 mg/dL (95% CI -7.83 to -0.24) | 0.03 | 0.001 |
| Biological sex | Female | 17 | WMD=-6.06 mg/dL (95% CI -7.58 to -4.53) | 0.001 | 0.2 |
|  | Male | 12 | WMD=-3.31 mg/dL (95% CI -6.14 to -0.47) | 0.02 | 0.001 |
|  | Female & male | 12 | WMD=-0.29 mg/dL (95% CI -0.84 to 0.26) | 0.3 | 0.001 |
| Degree of obesity (BMI) | Obesity | 25 | WMD=-1.2 mg/dL (95% CI -1.96 to -0.45) | 0.002 | 0.001 |
|  | Overweight | 16 | WMD=-3.54 mg/dL (95% CI -5.47 to -1.62) | 0.001 | 0.001 |
| Health status | Without other conditions | 33 | WMD=-3.04 mg/dL (95% CI -4.4 to -1.69) | 0.001 | 0.001 |
|  | With other conditions | 8 | WMD=-4.03 mg/dL (95% CI -7.54 to -0.51) | 0.02 | 0.001 |
| **Fasting insulin** | | | | | |
| Type of exercise | Aerobic | 25 | SMD=-0.79 (95% CI –1.1 to -0.47) | 0.001 | 0.001 |
|  | Combined | 10 | SMD=-1.0 (95% CI -1.53 to -0.46) | 0.001 | 0.001 |
|  | Resistance | 5 | SMD=-0.22 (95% CI -0.54 to 0.09) | 0.17 | 0.95 |
| Intervention duration | long-term >8 week | 32 | SMD=-0.81 (95% CI -1.1 to -0.52) | 0.001 | 0.001 |
|  | Short-term ≤8 week | 8 | SMD=-0.62 (95% CI -0.98 to -0.26) | 0.001 | 0.09 |
| Sex | Female | 18 | SMD=-1.2 (95% CI -1.63 to -0.78) | 0.001 | 0.001 |
|  | Male | 12 | SMD=-0.57 (95% CI -0.86 to -0.28) | 0.001 | 0.09 |
|  | Female & male | 10 | SMD=-0.26 (95% CI -0.54 to 0.007) | 0.05 | 0.1 |
| Degree of obesity (BMI) | Obesity | 24 | SMD=-0.87 (95% CI -1.2 to -0.55) | 0.001 | 0.001 |
|  | Overweight | 16 | SMD=-0.62 (95% CI -0.97 to -0.26) | 0.001 | 0.001 |
| Health status | Without other conditions | 32 | SMD=-0.67 (95% CI -0.93 to -0.42) | 0.001 | 0.001 |
|  | With other conditions | 8 | SMD=-1.12 (95% CI -1.66 to -0.59) | 0.001 | 0.001 |
| **HOMA-IR** | | | | | |
| Type of exercise | Aerobic | 23 | WMD=-0.76 (95% CI -1.03 to -0.5) | 0.001 | 0.001 |
|  | Combined | 9 | WMD=-1.11 (95% CI -1.71 to -0.51) | 0.001 | 0.001 |
|  | Resistance | 2 | WMD=-0.37 (95% CI -1.37 to 0.62) | 0.46 | 0.17 |
| Intervention duration | long-term >8 week | 27 | WMD=-0.79 (95% CI -1.06 to -0.53) | 0.001 | 0.001 |
|  | Short-term ≤8 week | 7 | WMD=-1.03 (95% CI -1.72 to -0.33) | 0.004 | 0.005 |
| Biological sex | Female | 14 | WMD=-1.23 (95% CI -1.58 to -0.87) | 0.001 | 0.001 |
|  | Male | 9 | WMD=-0.5 (95% CI -0.69 to -0.32) | 0.001 | 0.23 |
|  | Female & male | 11 | WMD=-0.27 (95% CI -0.66 to 0.11) | 0.16 | 0.01 |
| Degree of obesity (BMI) | Obesity | 21 | WMD=-0.99 (95% CI -1.36 to -0.62) | 0.001 | 0.001 |
|  | Overweight | 13 | WMD=-0.6 (95% CI -0.87 to -0.34) | 0.001 | 0.02 |
| Health status | Without other conditions | 26 | WMD=-0.69 (95% CI -0.93 to -0.44) | 0.001 | 0.001 |
|  | With other conditions | 8 | WMD=-1.45 (95% CI -2.16 to -0.74) | 0.001 | 0.001 |
| **Body weight** | | | | | |
| Type of exercise | Aerobic | 21 | WMD=-1.44 kg (95% CI -2.54 to -0.34) | 0.01 | 0.44 |
|  | Combined | 9 | WMD==-1.55 kg (95% CI -2.66 to -0.44) | 0.006 | 0.31 |
|  | Resistance | 4 | WMD=-0.61 kg (95% CI -3.74 to 2.5) | 0.69 | 0.02 |
| Intervention duration | long-term >8 week | 27 | WMD=-1.54 kg (95% CI -2.4 to -0.69) | 0.001 | 0.25 |
|  | Short-term ≤8 week | 7 | WMD=-1.55 kg (95% CI -4.33 to 1.21) | 0.27 | 0.09 |
| Biological sex | Female | 18 | WMD=-0.67 kg (95% CI -1.66 to 0.3) | 0.17 | 0.45 |
|  | Male | 8 | WMD=-3.83 kg (95% CI -5.22 to -2.45) | 0.001 | 0.97 |
|  | Female & male | 8 | WMD=-0.03 kg (95% CI -0.99 to 0.92) | 0.94 | 0.76 |
| Degree of obesity (BMI) | Obesity | 20 | WMD=-1.92 kg (95% CI -3.02 to -0.82) | 0.001 | 0.09 |
|  | Overweight | 14 | WMD=-0.38 kg (95% CI -1.52 to 0.76) | 0.51 | 0.39 |
| Health status | Without other conditions | 27 | WMD=-1.35 kg (95% CI -2.02 to -0.67) | 0.001 | 0.17 |
|  | With other conditions | 7 | WMD=0.6 kg (95% CI -0.93 to 2.14) | 0.44 | 0.49 |
